# Successful Right Upper Lung Segmentectomy after Esophagectomy: Utilization of 4K 3-Dimensional Endoscopy and Near-Infrared Fluorescence in High-Risk Surgery

**DOI:** 10.70352/scrj.cr.24-0144

**Published:** 2025-02-01

**Authors:** Masaya Otabe, Sayaka Yamada, Atsushi Kagimoto, Takeshi Mimura

**Affiliations:** 1 Department of General Thoracic Surgery, National Hospital Organization, Kure Medical Center and Chugoku Cancer Center, Kure, Hiroshima, Japan; 2 The Clinical Training Center, National Hospital Organization, Kure Medical Center and Chugoku Cancer Center, Kure, Hiroshima, Japan

**Keywords:** lung segmentectomy, esophagectomy, 4K 3-dimensional endoscopic system

## Abstract

**INTRODUCTION:**

Lung resection after open esophagectomy poses significant technical challenges, particularly when the reconstructed gastrointestinal tract is on the same side as the lung lesion. The advent of 4K 3-dimensional (3D) endoscopic systems with near-infrared (NIR) fluorescence imaging using indocyanine green (ICG) has improved the precision of thoracic surgeries. We present a case of successful right upper lung segmentectomy for primary lung cancer after open esophagectomy, utilizing a 4K 3D endoscopic system and NIR imaging.

**CASE PRESENTATION:**

An 85-year-old female with a history of open esophagectomy for esophageal cancer 19 years earlier and comorbidities, including aplastic anemia and diabetes mellitus, was referred for the evaluation of a growing lesion in the right upper lung. Computed tomography (CT) revealed a 43-mm tumor and the gastric tube, reconstructed during the prior esophagectomy, located in the right thoracic cavity. A CT-guided biopsy confirmed lung adenocarcinoma (cT2bN0M0, Stage IIA). Surgical challenges included severe adhesions from the previous thoracotomy and thrombocytopenia (platelet count: 20000) due to aplastic anemia. A thoracoscopic segmentectomy of the anterior segment of the right upper lobe was performed using a 4K 3D endoscopic system (TIPCAM1 Rubina; Karl Storz, Tuttlingen, Germany). Adhesions were meticulously dissected, and intraoperative platelet transfusions were administered. NIR imaging with ICG identified the intersegmental plane and confirmed blood flow to the gastric tube, preventing ischemic complications. The lung segmentectomy was completed using staplers, preserving the right gastroepiploic artery. Histopathology revealed acinar adenocarcinoma (pT3N0M0, Stage IIB). The patient resumed oral intake on postoperative Day 1 and was discharged on Day 13 without complications. No recurrence was noted during the follow-up.

**CONCLUSIONS:**

This case demonstrates the effective use of 4K 3D endoscopic systems and NIR imaging with ICG in complex lung resections following open esophagectomy. These technologies facilitate precise dissection and blood flow assessment, which are crucial for preserving reconstructed structures and enhancing surgical safety.

## Abbreviations

CT
: computed tomography
3D
: three-dimensional
NIR
: near-infrared
ICG
: indocyanine green
FDG
: ^18^F-fluorodeoxyglucose
VC
: vital capacity
FEV1
: forced expiratory volume in 1 s

## INTRODUCTION

Lung resection after esophagectomy, whether performed via a large thoracotomy or when the reconstructed gastrointestinal tract is located on the same side as the lung lesion, presents significant technical challenges. These procedures demand meticulous dissection to manage severe adhesions and preserve the reconstructed gastrointestinal tract. Recent advancements in three-dimensional (3D) endoscopic systems with 4K resolution, combined with near-infrared (NIR) fluorescence imaging using indocyanine green (ICG), have significantly enhanced surgical precision and performance.^1)^

Here, we report a successful case of right upper lung segmentectomy for primary lung cancer following open esophagectomy, where the reconstructed gastric tract was located in the right thoracic cavity, utilizing a 4K 3D endoscopic system and NIR imaging with ICG.

## CASE PRESENTATION

**Fig. 1:**
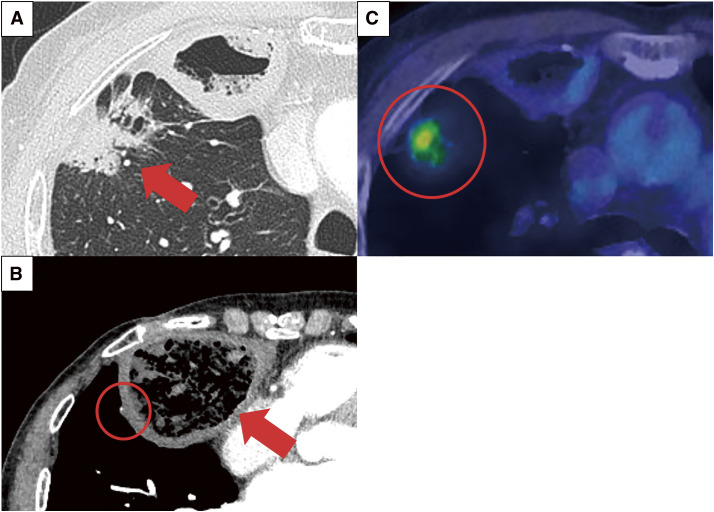
(**A**) Preoperative CT scan showing the tumor, measuring 43 mm at its largest dimension, located in the anterior segment of the right upper lobe (red arrow). (**B**) The reconstructed gastric tube protruding into the right thoracic cavity, positioned close to the right lung (red arrow) and its feeding vessel (red circle). (**C**) ^18^F-FDG positron emission tomography/CT demonstrating FDG uptake in the tumor with a maximum standardized uptake value of 4.0 (red circle). CT, computed tomography; FDG, fluorodeoxyglucose

An 85-year-old female with a history of open esophagectomy for esophageal cancer 19 years prior, and comorbidities including aplastic anemia and diabetes mellitus, was referred to our department due to the enlargement of a shadow in the upper lobe of the right lung. Computed tomography (CT) revealed a 43-mm lung tumor in the right upper lobe and a gastric tube, reconstructed during the esophagectomy and located in the anterior mediastinum, protruding into the right thoracic cavity (**Figs. 1A** and **1B**). ^18^F-fluorodeoxyglucose (FDG) positron emission tomography/CT demonstrated FDG accumulation in the tumor, with a maximum standardized uptake value of 4.0 (**Fig. 1C**). A CT-guided biopsy confirmed primary lung adenocarcinoma (cT2bN0M0, cStage IIA). Subsequently, lung segmentectomy of the right anterior segment (S3) was chosen due to the patient’s advanced age and the tumor size, which rendered partial resection unsuitable. Additionally, a right upper lobectomy was avoided due to the need for extensive posterior dissection, which posed a risk of tracheal necrosis.^2)^ There were several concerns for performing radical surgery for lung cancer in this patient. First, the patient had a history of esophagectomy performed via large thoracotomy, which suggests the presence of intrathoracic adhesions. Furthermore, the reconstructed gastric tube in the anterior mediastinum was located within the right thoracic cavity. Contrast-enhanced CT showed that the right gastroepiploic artery, which is a feeding vessel, was in close proximity to the right upper lung lobe that was to be resected (**Fig. 1B**). Second, the patient had severe thrombocytopenia due to aplastic anemia, with a critically low platelet count (approximately 20000). Although alternative treatments such as radiotherapy were considered because of the associated risks, the large size and unclear margins of the tumor, as well as the proximity of the reconstructed gastric tube to the radiation field, presented substantial challenges. The preoperative pulmonary function test demonstrated that the vital capacity (VC) was measured at 2430 mL, with the predicted VC of 99.1%. The forced expiratory volume in 1 s (FEV1) was 1840 mL, with the ratio of FEV1 to the forced VC being 79.3%, and the predicted diffusing capacity of carbon monoxide was 107.6%. Additionally, transthoracic echocardiography revealed good cardiac contractility (ejection fraction: 60%) with no regional wall motion abnormalities. These findings, combined with the patient’s well-preserved organ function and a high level of activity of daily living, suggesting suitability for surgery. Consequently, we chose lung resection to achieve complete tumor removal. The patient was transfused 200 mL of platelets on the eve and day of surgery, following the guideline stating that a platelet count of ≥50000/μL is required for operability.^3)^ On the morning of the surgery, the platelet count was 60000/μL, and the operation was deemed feasible.

**Fig. 2:**
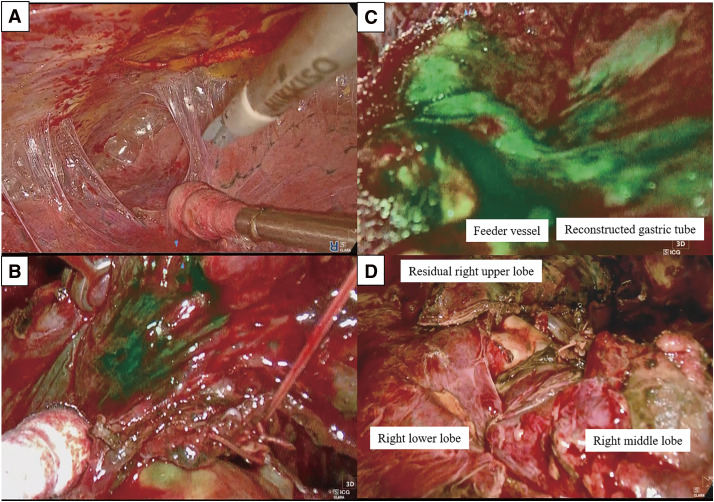
View of the lung segmentectomy of the anterior segment of the right upper lobe. (**A**) Severe adhesions observed in the thoracic cavity. (**B**) The intersegmental plane on the mediastinal side of the right upper lobe clearly delineated using NIR fluorescence imaging with ICG. (**C**) Blood flow in the reconstructed gastric tube and its feeding vessels assessed using NIR fluorescence imaging with ICG, confirming the absence of ischemia in the reconstructed gastric tube. (**D**) The intrathoracic view after completion of the right anterior segmentectomy utilizing a 4K 3D endoscopic system. NIR, near-infrared, ICG, indocyanine green, 3D, three-dimensional

The operation was performed with platelet transfusions and a nasogastric tube was inserted for gastric decompression. Using a 4K 3D endoscopic system (TIPCAM1 Rubina; Karl Storz, Tuttlingen, Germany), we initiated the operation thoracoscopically through four ports. Severe thoracic adhesions, especially near the prior thoracotomy site, which required careful dissection, were present (**Fig. 2A**). Significant oozing due to thrombocytopenia was controlled using soft coagulation, SURGICEL Powder Absorbable Hemostat (Ethicon, New Brunswick, NJ, USA), and TachoSil (CSL Behring, King of Prussia, PA, USA). The reconstructed gastric tube and the adipose tissue containing the right gastroepiploic artery were identified and preserved. At the pulmonary hilum, branches of the pulmonary artery and vein to the anterior segment were ligated and divided. The bronchus to the anterior segment was divided using a surgical stapler. ICG (12.5 mg) was intravenously injected, and the planned intersegmental plane line was visualized under NIR imaging (**Fig. 2B**). The mediastinal side of the segment was clearly delineated, although visualization of the lateral side was hindered by hemorrhage due to thrombocytopenia. Simultaneously, blood flow in the reconstructed gastric tube and its feeding vessels was assessed, confirming the absence of ischemia (**Fig. 2C**). Segmentectomy was completed using surgical staplers along the intersegmental plane (**Fig. 2D**, **Supplementary Video**). The operation lasted 401 minutes with an intraoperative blood loss of 310 mL, and the volume of platelets transfused intraoperatively was 200 mL. Pathological examination revealed acinar adenocarcinoma with an invasive size of 30 mm × 20 mm × 30 mm and with the invasion of the parietal pleura (pT3N0M0, pStage IIB); the surgical margin was 15 mm. The patient resumed oral intake the day after surgery and experienced no complications, including issues with the reconstructed gastric tube. The patient was discharged on postoperative Day 13, and no recurrence was observed during follow-up visits.

## DISCUSSION

Anatomical lung resection for ipsilateral lung cancer following open thoracotomy after esophagectomy poses significant technical challenges due to severe adhesions and the need to preserve the reconstructed gastrointestinal tract. In this case, after esophagectomy with posterolateral thoracotomy, the reconstructed gastric tube, which was intended to be positioned behind the sternum, had migrated into the right thoracic cavity. Extensive adhesions were observed, not only between the thoracotomy site and the lung but also between the reconstructed gastric tube and the lung targeted for resection. Using a 4K 3D endoscopic system and assessing blood flow with NIR fluorescence imaging with ICG, we successfully performed the complex segmentectomy without complications.

Advances in chemotherapy, surgical techniques, and perioperative care have significantly improved the prognosis of patients with esophageal cancer.^4–6)^ However, due to shared risk factors such as smoking,^7)^ cases requiring lung resection after esophagectomy are becoming more common. As Watanabe^8)^ noted, lung resection after esophagectomy carries a heightened risk of complications, particularly aspiration pneumonia. They highlight the importance of fasting management before lung resection to prevent the risk of intraoperative aspiration. Additionally, lung resection ipsilateral to the esophagectomy and lobectomy were found to be significant predictors of postoperative morbidity. Delicate intraoperative handling is crucial, especially in cases like this, where the reconstructed gastrointestinal tube—intended to reside in the anterior mediastinum behind the sternum—was instead located in the right thoracic cavity. The presence of thrombocytopenia due to aplastic anemia further required extremely precise dissection of dense intrathoracic adhesions to minimize hemorrhage and preserve the reconstructed gastric tube. Under these challenging circumstances, the 4K 3D endoscopic system proved invaluable. Although the procedure using HD 2D endoscopic systems might be feasible, the 3D visualization offered by the 4K 3D system provided superior depth perception and higher-definition images, facilitating the accurate identification of structures. Reportedly, 3D endoscopic systems contribute to shorter operative times and reduced intraoperative blood loss compared with 2D systems.^9)^ Furthermore, the 4K 3D system, with its superior resolution, has demonstrated additional advantages over HD 3D systems.^1)^ These benefits highlight the system value, particularly in challenging cases such as the present one.^9)^

In this case, the ability to assess blood flow in the reconstructed gastric tube during surgery, after dissecting severe adhesions, was a critical factor in preventing postoperative gastric tube necrosis. Preoperative CT revealed that the right gastroepiploic artery, which supplies the reconstructed gastric tube, was located in close proximity to the right upper lobe of the lung to be resected. The use of NIR imaging with ICG to evaluate blood flow after dissection provided reassurance and facilitated the successful completion of the surgery. In general thoracic surgery, NIR imaging with ICG is frequently used, particularly for identifying the intersegmental plane during lung segmentectomy.^10)^ Additionally, several studies have reported its utilities in evaluating blood flow in reconstructed gastric tubes and colonic grafts after esophagectomy.^11)^ To our knowledge, this is the first reported case of lung segmentectomy in a patient with a history of open esophagectomy in which the 4K 3D endoscopic system and NIR imaging were both utilized. While similar applications have been reported in liver resection,^12)^ their use in other fields remains limited, highlighting the potential of emerging technologies for enhancing the safety and feasibility of performing challenging thoracic surgical procedures.

## CONCLUSION

This case demonstrates the effective use of 4K 3D endoscopic systems and NIR imaging with ICG for complex lung resections post open esophagectomy. These advanced technologies facilitate precise dissection and accurate blood flow assessment, which are crucial for preserving the integrity of reconstructed structures and enhancing surgical safety.

## SUPPLEMENTARY MATERIALS

Supplementary Video A video clip showing the lung segmentectomy of the anterior segment of the right upper lobe.

## ACKNOWLEDGMENTS

We would like to thank Enago (https://www.enago.jp/) for English language editing.

## DECLARATIONS

### Funding

This study received no financial support.

### Authors’ contributions

MO drafted the manuscript.

AK and TM supervised the final manuscript.

All authors discussed the results and contributed to the final version of the manuscript.

All authors have read and approved the manuscript, and they are responsible for the manuscript.

### Availability of data and materials

The data supporting the findings of this study are available upon request from the corresponding author. The data are not publicly available due to privacy and ethical restrictions.

### Ethics approval and consent to participate

This case report was approved by the institutional review boards of Kure Medical Center and Chugoku Cancer Center (No. 2024-62).

### Consent for publication

Written informed consent was obtained from the patient for the publication of this case report and accompanying images.

### Competing interests

The authors declare no competing interests.
